# Leucine supplementation improves adiponectin and total cholesterol concentrations despite the lack of changes in adiposity or glucose homeostasis in rats previously exposed to a high-fat diet

**DOI:** 10.1186/1743-7075-8-62

**Published:** 2011-09-07

**Authors:** Francisco L Torres-Leal, Miriam H Fonseca-Alaniz, Gabriela FR Teodoro, Mariana D de Capitani, Daiana Vianna, Lucas C Pantaleão, Emidio M Matos-Neto, Marcelo M Rogero, Jose Donato, Julio Tirapegui

**Affiliations:** 1 Department of Food Science and Experimental Nutrition, Faculty of Pharmaceutical Sciences, University of São Paulo, São Paulo, Brazil; 2 Heart Institute (InCor), University of Sao Paulo Medical School, Sao Paulo, Brazil; 3 Department of Nutrition, School of Public Health, São Paulo University, Sao Paulo, Brazil; 4 Department of Internal Medicine, Division of Hypothalamic Research, University of Texas Southwestern Medical Center, Dallas, TX 75390 - USA

**Keywords:** Leucine, Adipose tissue, Endurance training, Body composition, Obesity, Adiponectin

## Abstract

**Background:**

Studies suggest that leucine supplementation (LS) has a therapeutic potential to prevent obesity and to promote glucose homeostasis. Furthermore, regular physical exercise is a widely accepted strategy for body weight maintenance and also for the prevention of obesity. The aim of this study was to determine the effect of chronic LS alone or combined with endurance training (ET) as potential approaches for reversing the insulin resistance and obesity induced by a high-fat diet (HFD) in rats.

**Methods:**

Forty-seven rats were randomly divided into two groups. Animals were fed a control diet-low fat (*n = *10) or HFD (*n = *37). After 15 weeks on HFD, all rats received the control diet-low fat and were randomly divided according to treatment: reference (REF), LS, ET, and LS+ET (*n = *7-8 rats per group). After 6 weeks of treatment, the animals were sacrificed and body composition, fat cell volume, and serum concentrations of total cholesterol, HDL-cholesterol, triacylglycerol, glucose, adiponectin, leptin and tumor necrosis factor-alpha (TNF-α) were analyzed.

**Results:**

At the end of the sixth week of treatment, there was no significant difference in body weight between the REF, LS, ET and LS+ET groups. However, ET increased lean body mass in rats (*P *= 0.019). In addition, ET was more effective than LS in reducing adiposity (*P *= 0.019), serum insulin (*P *= 0.022) and TNF-α (*P *= 0.044). Conversely, LS increased serum adiponectin (*P *= 0.021) levels and reduced serum total cholesterol concentration (*P *= 0.042).

**Conclusions:**

The results showed that LS had no beneficial effects on insulin sensitivity or adiposity in previously obese rats. On the other hand, LS was effective in increasing adiponectin levels and in reducing total cholesterol concentration.

## Background

Obesity is associated with a number of health problems that are often summarized as the metabolic syndrome, and its etiology may be associated with the consumption of high energy-dense foods [1]. Excessive intake of dietary fat promotes adipocyte hypertrophy, altering their normal endocrine function to an inflammatory pathologic condition that increases the secretion of tumor necrosis factor-alpha (TNF-α) and interleukin-6 (IL-6), among other proinflammatory cytokines, and concomitantly reducing adiponectin secretion [2,3].

Previous studies have demonstrated that dietary leucine regulates body weight and glucose homeostasis [4-10]. Within this context, several studies support the hypothesis that leucine plays an important role in the regulation of metabolism and energy balance by directly affecting peripheral tissues, such as white adipose tissue, liver, and muscle. For example, leucine has been shown to increase the secretion of leptin [11] and adiponectin [12,13] from adipocytes.

Another important and widely accepted strategy for the maintenance of body weight and prevention of obesity is regular physical exercise [14,15]. The beneficial effects of physical exercise on weight loss are attributed to body fat reduction, maintenance of lean body mass and improvement of metabolic condition, e.g., increasing glucose uptake, especially in skeletal muscle [14,15]. Moreover, chronic physical exercise has several effects secondary to body fat loss, including a reduction in the synthesis and secretion of proinflammatory adipokines [16,17] and an increase in circulating levels of adiponectin, the main anti-inflammatory molecule secreted by adipocytes [18,19]. Physical exercise increases energy expenditure, exerting a greater effect during a state of negative energy balance, especially when combined with a reduction in the consumption of high energy-dense foods [20].

Within this context, in which the most recommended lifestyle changes for the treatment of obesity and diabetes include an adequate diet and regular physical exercise, leucine supplementation (LS) and physical exercise may affect the maintenance of body weight and, possibly, the metabolic status. Therefore, the present study was designed to evaluate the potential therapeutic effect of these two factors on the treatment of obesity. For this purpose, adult rats previously treated for 15 weeks with a high-fat diet (HFD) received chronic LS alone or combined with endurance training (ET). The effects of 6-week LS on body composition, food intake, adipocyte volume, and serum levels of several pro- and anti-inflammatory metabolic markers were assessed.

## Methods and Procedures

### Animals

Male Sprague-Dawley rats (approximately 120 days old) were obtained from the Animal Laboratory of the Faculty of Pharmaceutical Sciences at the University of São Paulo and were kept with food and water ad libitum. The rats were maintained in a room at an ambient temperature of 22 ± 2°C and a relative humidity of 55 ± 10% under a 12-h light/12-h dark cycle. All animal procedures were approved by the Ethics Committee for animal experimentation of the Faculty of Pharmaceutical Sciences, University of São Paulo, according to the guidelines of the Brazilian College on Animal Experimentation.

### Diet and experimental design

The experimental diets were prepared according to the recommendations of the American Institute of Nutrition for adult rats and are shown in Table 1[21]. After weight distribution, rats were fed the control diet (CD, *n = *10) or HFD (*n = *37) for 15 weeks. At the end of the first 15 weeks, 10 animals in the CD group and 7 animals in the HFD group were sacrificed in order to evaluate their metabolic status. After 15 weeks on HFD, the obese animals were divided into four subgroups of treatment for 6 weeks: i) reference (REF) group (REF, *n = *7) receiving the CD; ii) LS group (LS, *n = *8) receiving diet supplemented with 5% L-leucine (Ajinomoto Interamericana Indústria e Comércio Ltda, São Paulo, Brazil); iii) endurance training group (ET, *n = *8) receiving the CD, and iv) LS plus ET group receiving diet supplemented with 5% L-leucine (LS+ET, *n = *7). Other studies used this leucine dose and no toxic effect was reported [22,23].

**Table 1:** Diet composition^1^

	Control diet	High-fat diet	+ Leucine diet
		*g/kg diet*	
Cornstarch	620.6	282.6	570.6
Casein	140.0	140.0	140.0
Sucrose	100.0	100.0	100.0
Soybean oil	40.0	40.0	40.0
Lard	-	338.0	-
Cellulose	50.0	50.0	50.0
AIN-93M mineral mixture	35.0	35.0	35.0
AIN-93M vitamin mixture	10.0	10.0	10.0
Choline bitartrate	2.5	2.5	2.5
L-Cystine	1.8	1.8	1.8
tert-Butylhydroquinone	0.008	0.008	0.008
L-Leucine	-	-	50.0
Total (g)	1.000	1.000	1.000

^1^Based on AIN-93M (21).

Body weight and food intake were assessed weekly and final body weight was recorded immediately before euthanasia. At the end of 21 weeks, rats were fasted for 12 hours, and 72 hours after the last exercise session, the animals were anesthetized with rodent cocktail [xylazine hydrochloride (20 mg/mL), ketamine hydrochloride (100 mg/mL), acepromazine (20 mg/mL), and distilled water (4.5/4.5/1.8/7.2, v/v)]. Trunk blood samples were collected after decapitation and centrifuged and serum was stored in a freezer at -80°C. The gastrointestinal tract was completely emptied and washed with saline. Subcutaneous (SC), epididymal (EP), and retroperitoneal (RP) white adipose fat pads were totally excised, weighed, and processed for adipocyte isolation. Body composition was determined by chemical analysis of the carcass as previously described by Donato et al. [5].

### Endurance training

The exercise program consisted of continuous swimming in individual tanks filled with water and maintained at 28-32°C. Animals were trained for 6 weeks during 60-min daily sessions, five times a week. All rats swam with a load of 5% body weight, which was attached to their tail. This training protocol is already standardized in our laboratory [24,25].

### Adipocyte isolation

Adipocytes were isolated with collagenase as previously described [26]. The isolated adipocytes (~7 to 8 × 10^5 ^cells/mL) were resuspended in EHB buffer (Earle's salts, 20 mM HEPES, 1% bovine serum albumin, 2 mM sodium pyruvate, and 4.8 mM sodium bicarbonate), pH 7.4 at 37°C. For morphometric analysis, aliquots of the cell suspension were used to measure the transverse diameter of 100 isolated adipocytes with a light microscope equipped with a micrometer. Assuming that the isolated adipocyte is spherical, this value was used to calculate the volume and average cell surface area according to the formulas proposed by Fine & Digirolamo [27]. The number of adipocytes was determined by the ratio of total tissue weight (pg) to average adipocyte mass (pg).

### Serum analysis

Serum levels of total cholesterol, high-density lipoprotein (HDL)-cholesterol, triacylglycerol (TG), and glucose were determined by enzymatic methods using commercial kits (Labtest Diagnóstica kit, Glucose PAP Liquiform, São Paulo, Brazil). Serum leptin and insulin concentrations were quantified using radioimmunoassay kits (Linco Research, Inc., St Charles, MO, USA). Serum adiponectin level was determined with an enzyme-linked immunosorbent assay (ELISA) kit (Linco Research, Inc.). Serum TNF-α d was quantified using the LINCOplex assay (Linco Research, Inc.). The blood concentration of amino acids was determined by the methods of White et al. [28] and Hagen et al. [29]. The homeostasis model assessment (HOMA) index was calculated as an indicator of insulin resistance: HOMA = (fasting plasma insulin concentration (ng/mL)) × (fasting plasma glucose (mmol/L))/22.5

The ratio adiponectin/fat pad weight ratio was calculated by dividing the basal adiponectin for fat pad weight.

### Statistical analysis

Data are expressed as mean ± SEM. The unpaired two-tailed Student *t *test was used to evaluate obesity status. The effect of LS and ET was analyzed by factorial ANOVA (2^2^) followed by Tukey's honestly significant difference test. ANOVA for repeated measures was used for the comparison of body weight, followed by Dunnett's test when comparing times. Differences were considered to be significant when *P *≤ 0.05. Calculations were performed and graphs were drawn using the Statistica version 7.1 program (StatSoft).

## Results

### Characterization of obesity model, hyperglycemia and hyperinsulinemia

Administration of the HFD for 15 weeks induced a significant increase in total adiposity (*P *= 0.024), including increases in SC (*P *= 0.045), EP (*P *= 0.004) and RP (*P <*0.001) fat pads and hypertrophy of SC (*P *= 0.041) and EP (*P *= 0.014) adipocytes when compared to the CD group (Table 2).

**Table 2:** Obesity state and metabolic parameters in rats after 15 weeks of dietary treatment

	Control diet	High-fat diet	*P*
Body fat, *%*	14.46 ± 1.16	20.94 ± 2.39*	0.024
SC fat pad, *g/100 g*	2.77 ± 0.17	4.09 ± 0.69*	0.045
EP fat pad, *g/100 g*	1.98 ± 0,14	2.72 ± 0.17*	0.004
RP fat pad, *g/100 g*	1.40 ± 0.09	2.29 ± 0.19*	0.000
EP adipocyte volume, *pL*	350.80 ± 22.42	432.80 ± 14.28*	0.014
SC adipocyte volume, *pL*	257.90 ± 28.31	359.30 ± 36.55*	0.041
Glucose, *mg/dL*	132.91 ± 4.73	148.80 ± 3.88*	0.035
Insulin, *ng/mL*	0.53 ± 0.04	0.99 ± 0.19*	0.022
TNF-α, *pg/mL*	45.78 ± 2.54	59.05 ± 3.94*	0.010
Leptin, *ng/mL*	12.70 ± 1.22	25.40 ± 2.78*	0.000
Adiponectin, *ng/mL*	22.67 ± 1.38	16.38 ± 1.28*	0.006

SC, subcutaneous; EP, epididymal; RP, retroperitoneal

Values are mean ± sem (control group, *n = *10; high-fat group, *n = *7)

*P <0.05 vs. control diet group.

We also observed a significant increase in blood glucose (12%) (*P = *0.035), insulin (86%) (*P *= 0.022), TNF-α (28%) (*P *= 0.010), and leptin (130%) (*P *< 0.001) levels in the HFD group compared to the CD group. On other hand, serum adiponectin concentration was significantly (*P *= 0.006) reduced (-27%) in the HFD group (Table 2). Taken together, these results indicate that the HFD caused obesity and insulin resistance and altered the blood levels of major adipokines in rats.

### LS and ET in the treatment of diet-induced obesity

**Figure 1:**
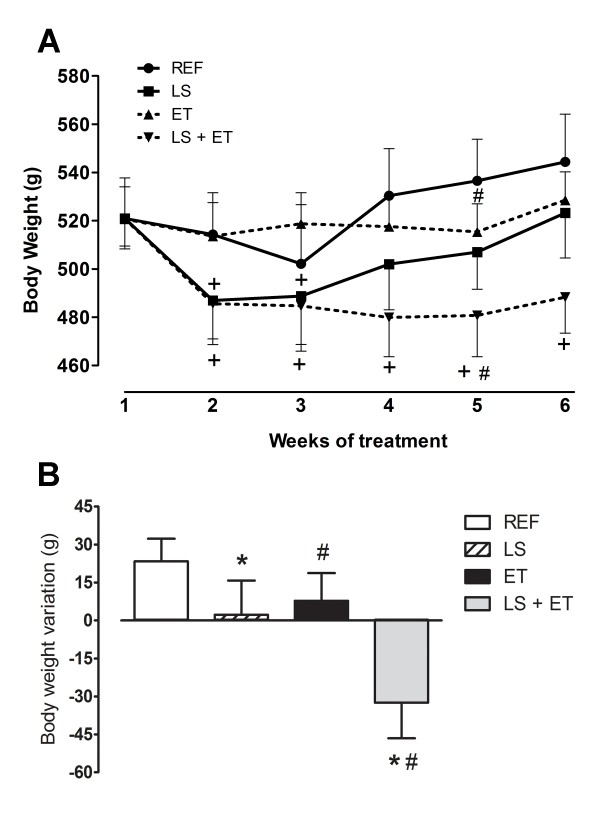
**Effect of chronic exercise training and/or leucine supplementation on body weight changes**. (A) Body weight. ^#^, effect of training in fifth week (*P *= 0.045); + *vs *week 1 (*P *< 0.002). (B) Total body weight variation (final - initial body weight). *, effect of diet (*P = *0.028); ^#^, effect of training, (*P = *0.006). Experimental groups: control diet (REF), diet supplemented with 5% L-leucine (LS), 6 weeks of endurance training (ET) plus standard diet, and diet supplemented with 5% L-leucine plus endurance training (LS+ET). Values are mean ± sem (*n *= 7-8 rats per group).

After induction of obesity with HFD, the groups of rats started the treatments with a similar initial body weight. No significant differences in daily energy intake were observed between groups: REF (90.26 ± 9.37 kcal/day); LS (97.75 ± 7.51 kcal/day); ET (98.75 ± 10.44 kcal/day); LS+ET (82.26 ± 11.19 kcal/day); diet (*P *= 0.655); training (*P *= 0.728), and interaction (*P *= 0.252). During the fifth week of the experiment, physical training significantly (*P *= 0.045) reduced body weight. However, at the end of the sixth week of treatment, there was no significant difference (*P *> 0.05) in body weight between the REF, LS, ET and LS+ET groups (Figure 1A). In addition, leucine exerted a transient effect on the reduction of body weight in the LS group in the second and third week (*P = *0.002). In parallel, LS+ET caused weight loss that persisted throughout the experimental period (*P <*0.001) (Figure 1A). When body weight variation was analyzed, both LS (*P *= 0.028) and ET (*P *= 0.006) significantly reduced body weight gain. Nevertheless, analysis of treatment interaction showed similar responses in body weight variation for the two treatments (Figure 1B).

### ET is more efficient than LS in reducing body adiposity in previously obese rats

**Figure 2:**
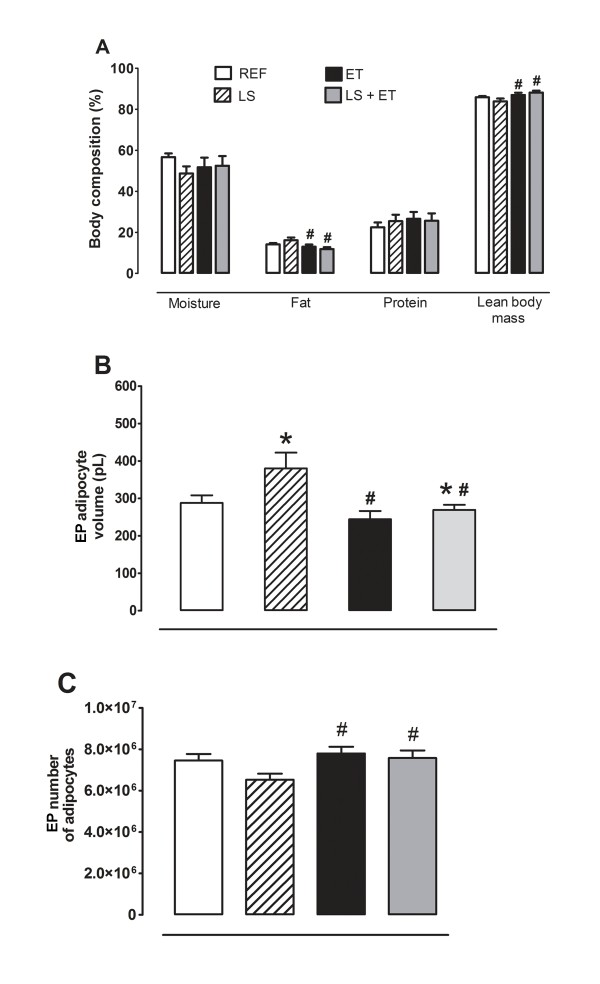
**Chronic exercise is more efficient than leucine supplementation in reducing adiposity**. (A) Body composition (%). ^#^, effect of training (*P *< 0.019). (B) Epididymal adipocyte volume. ^#^, effect of training (*P *< 0.005); *, effect of diet, (*P <*0.020). (C) Epididymal adipocyte number ^#^, effect of training (*P *< 0.044). Experimental groups: control diet (REF), diet supplemented with 5% L-leucine (LS), 6 weeks of endurance training (ET) plus standard diet, and diet supplemented with 5% L-leucine plus endurance training (LS+ET). Values are mean ± sem (*n *= 7-8 rats per group).

ET reduced (*P *= 0.019) fat content in the carcass (Figure 2A). Furthermore, we found higher lean mass in trained animals compared to sedentary animals (*P *= 0.019). However, there was no significant difference (*P *> 0.05) in carcass protein or moisture content in trained animals supplemented with leucine and no interaction between these variables was observed (Figure 2A).

EP fat pad weight was significantly (*P *= 0.033) lower in the trained group compared to the sedentary group (Table 3). Conversely, LS was less effective in reducing body fat as can be seen by the higher (*P *= 0.030) value obtained for the sum of fat pads (Table 3). Both LS (*P *= 0.020) and ET (*P *= 0.005) induced distinct but significant effects on EP adipocyte volume (Figure 2B). In parallel, LS was less effective in reducing adipocyte expansion in the EP region, whereas ET significantly reduced EP adipocyte volume. Furthermore, ET increased the number of EP adipocytes (*P *= 0.044) (Figure 2C).

**Table 3:** Effect of chronic (6 weeks) physical training and/or leucine supplementation on fat pad weight

					2-Way ANOVA P-values		
					
	REF	LS	ET	LS+ET	Diet	Training	Interaction
SC, *g/100 g b.w*.	2.72 ± 0.20	3.23 ± 0.35	2.59 ± 0.21	2.92 ± 0.18	0.110	0.397	0.717
EP, *g/100 g b.w*.	1.93 ± 0.16	2.45 ± 0.29	1.70 ± 0.14	1.82 ± 0.07	0.108	0.033*	0.301
RP, *g/100 g b.w*.	1.36 ± 0.15	1.78 ± 0.23	1.18 ± 0.13	1.41 ± 0.09	0.054	0.095	0.554
∑, *g/100 g b.w*.	6.01 ± 0.45	7.79 ± 0.82	5.47 ± 0.45	6.14 ± 0.30	0.030*	0.052	0.305

(REF), control diet; (LS), diet supplemented with 5% L-leucine; (ET), endurance training plus control diet; (LS+ET), diet supplemented with 5% leucine plus endurance training.

SC, subcutaneous; EP, epididymal; RP, retroperitoneal; **∑**, sum of fat pad weight.

Values are mean ± sem (*n *= 7-8 rats per group).

*Effect of diet, *P <*0.05; #effect of training, *P *< 0.05.

### LS reduces serum total cholesterol levels in sedentary animals

**Figure 3:**
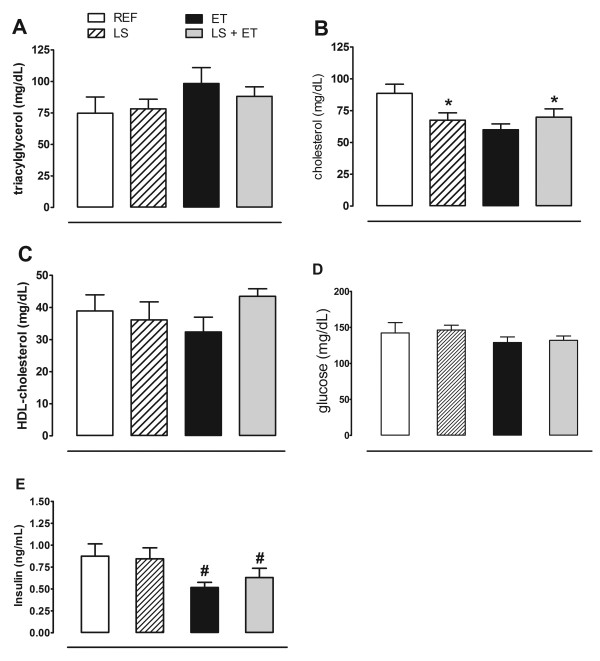
**Leucine supplementation reduces serum total cholesterol levels in sedentary animals**. (A) Serum concentration of triacylglycerol. (B) Serum concentration of cholesterol. *, effect of diet (*P <*0.042); interaction of the two factors (*P *= 0.018). (C) Serum concentration of HDL-cholesterol. (D) Serum concentration of glucose. (E) Serum concentration of Insulin. ^#^, effect of training (*P *< 0.022). Experimental groups: control diet (REF), diet supplemented with 5% L-leucine (LS), 6 weeks of endurance training (ET) plus standard diet, and diet supplemented with 5% L-leucine plus endurance training (LS+ET). Values are mean ± sem (*n *= 5-8 rats per group).

Both LS and ET did not induce significant differences in serum TG (Figure 3A) or HDL-cholesterol levels (Figure 3C). LS reduced serum total cholesterol concentration in sedentary animals (*P *= 0.042), but not in trained animals, since a significant difference was only observed for the interaction of the two factors (*P *= 0.018) (Figure 3B).

### ET is more efficient than LS in reducing insulin levels and HOMA index

Serum glucose level was not significantly altered by diet or ET (Figure 3D). However, only ET significantly reduced (*P *= 0.022) serum insulin concentration (Figure 3E). Regarding HOMA index, a strong trend (*P *= 0.059) in reducing insulin resistance status was seen in the trained animals when compared with sedentary groups, REF (0.29 ± 0.05); LS (0.32 ± 0.06); ET (0.21 ± 0.05); LS+ET (0.20 ± 0.03); diet (*P *= 0.872); training (*P *= 0.059), and interaction (*P *= 0.683).

### LS and ET differently affect the levels of adipokines

**Figure 4:**
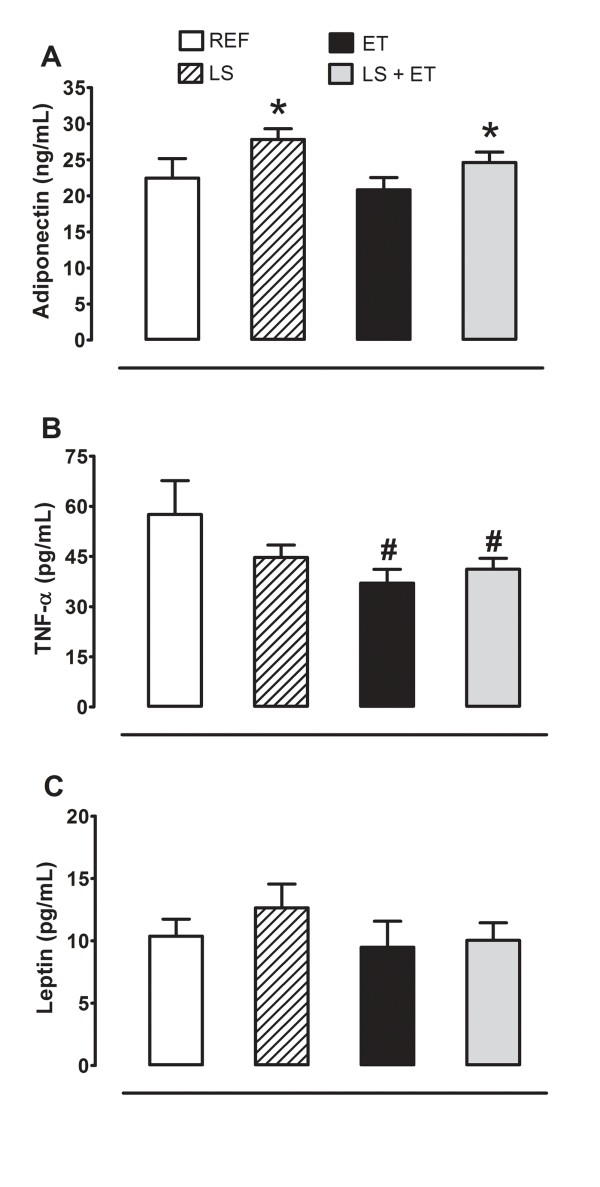
**Leucine supplementation increases adiponectin levels**. (A) Adiponectin. *, effect of diet (*P <*0.021). (B) TNF-α. ^#^, effect of training (*P *< 0.044). (C) Leptin. Experimental groups: control diet (REF), diet supplemented with 5% L-leucine (LS), 6 weeks of endurance training (ET) plus standard diet, and diet supplemented with 5% L-leucine plus endurance training (LS+ET). Values are mean ± sem (*n *= 6-8 rats per group).

Serum adiponectin level was significantly increased (*P *= 0.021 for diet factor) after 6 weeks of LS (Figure 4A), but the same effect was not observed for ratio adiponectin/fat pad weight, REF (3.81 ± 0.43 ng/mL/100 g); LS (5.56 ± 0.75 ng/mL/100 g); ET (3.66 ± 0.39 ng/mL/100 g); LS+ET (4.08 ± 0.33 ng/mL/100 g); diet (*P *= 0.041); training (*P *= 0.121), and interaction (*P *= 0.202) and TNF-α (Figure 4B). ET was effective in significantly reducing (*P *= 0.044) TNF-α concentration when compared to the sedentary groups (Figure 4B), whereas serum leptin concentration was not significantly affected by either diet or ET (Figure 4C).

### Effect of LS and ET on the plasma concentration of amino acids

**Figure 5:**
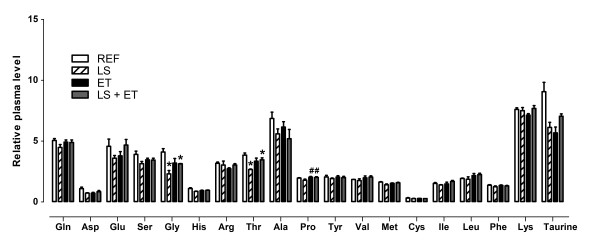
**Effect of chronic exercise and/or leucine supplementation on circulating levels of amino acids**. ^#^, effect of training (*P *< 0.05); *, interaction for amino acids glycine and threonine (*P <*0.05). Experimental groups: control diet (REF), diet supplemented with 5% L-leucine (LS), 6 weeks of endurance training (ET) plus standard diet, and diet supplemented with 5% L-leucine plus endurance training (LS+ET). Values are mean ± sem (*n *= 5-4 rats per group).

Plasma leucine concentration was not significantly altered by diet (*P *= 0.952) or ET (*P *= 0.077) in overnight fasted rats. Moreover, significant differences were observed for some amino acids after overnight fasting, with the effects related to supplementation with leucine [glycine, (*P *= 0.008) and threonine, (*P *= 0.015)], ET [proline, (*P *= 0.038)] and we found some interactions [aspartic acid, (*P *= 0.018), glycine (*P *= 0.012), histidine (*P *= 0.031), threonine (*P *= 0.005), methionine (*P *= 0.033) and taurine (*P *= 0.003)] (Figure 5).

## Discussion

Previous studies have demonstrated improvement in the control of body weight or a reduction of body fat content with leucine or branched-chain amino acid supplementation [5,8,30-32]. However, other studies have not observed these responses [4,9,33]. In some previous studies, animals were simultaneously exposed to a HFD and LS. Thus, these studies investigated the effect of leucine on the prevention of diet-induced obesity [8,9]. In our study, we first induced obesity, hyperglycemia and hyperinsulinemia in animals using a HFD. After establishment of diet-induced obesity, the animals received a control diet supplemented or not with leucine in order to study the potential of this amino acid in reverting the obesity, hyperglycemia and hyperinsulinemia phenotypes associated with long-term exposure to a HFD. Furthermore, we evaluated the effects of LS combined with ET, since physical exercise is a recognized strategy used to improve body weight control and insulin sensitivity. We found that chronic LS in this experimental paradigm did not alter food intake nor did it reduce fat pad mass or visceral adipocyte volume. We also showed that LS affected the endocrine function of adipocytes, increasing the circulating levels of adiponectin. Furthermore, we observed a beneficial effect of LS on the serum concentrations of total cholesterol. However, no changes in serum glucose, insulin, leptin, or TNF-α were seen. In comparison to LS alone, ET was more effective in attenuating the degree of adiposity, hyperinsulinemia and circulating levels of TNF-α.

The model of obesity induced by HFD is widely accepted and has been used alone, or in combination with other strategies, as an animal model of excess body fat [1,34,35]. Our results validate the diet employed to induce obesity, confirming that rats chronically exposed to a HFD present elevated body fat with hypertrophy of SC and EP adipocytes, leptin resistance, abnormal glucose homeostasis and a proinflammatory state characterized by increased serum TNF-α and reduced serum adiponectin levels. Therefore, LS was evaluated in animals already showing indicators of metabolic syndrome [1,34,35].

The present results indicate at transient effect of LS on body weight, since we observed a lower body weight at the beginning of supplementation. However, these animals recovered their weight later. Under these experimental conditions, leucine is unable to maintain its effect of reducing body weight for a prolonged period of supplementation. On the other hand, when supplementation is combined with ET body, weight loss is equally achieved and maintained during the treatment period.

With regard to adipose mass and adipocyte volume, we observed independent effects of LS and ET. In this case, whereas trained animals displayed reduced adiposity, leucine-treated animals had greater fat mass and fat cell volume. The present results regarding adiposity agree with the already known heterogeneity of fat pads. In this respect, LS favored a higher adiposity in the visceral region, irrespective of the fat pad assessed. Interestingly, ET affected the same fat pads, but in opposite way, favoring fat mass reduction.

This effect on adiposity observed in leucine-treated animals may be related to mammalian target of rapamycin (mTOR) activation. Although we did not directly assess mTOR activation in adipose tissue, our results are consistent with those obtained by Chakrabarti et al. [36]. These authors showed that activation of mTORC1 signaling in 3T3-L1 adipocytes transcriptionally inhibits the expression of adipose triglyceride lipase (ATGL) and hormone sensitive lipase (HSL), thus suppressing lipolysis of TG and diacylglycerol in adipose tissue. Moreover, these authors found that mTORC1 activation in adipocytes promotes de novo lipogenesis and TG accumulation. Accordingly, Polak et al. [37] observed that adipocytes from knockout animals for raptor, a regulatory protein associated with rapamycin-sensitive mTOR and responsible for mTORC1 activity [38], had lower deposition of TG. In a recent study, Zeanandin et al. [23] demonstrated that 18-month-old rats showed hypertrophy and hyperplasia of adipose tissue after 6 months of LS. These effects were attributed to an increased phosphorylation of mTOR and increased mRNA expression of PPARγ in adipose tissue. This evidence suggests that mTOR signaling is an important intracellular pathway, directly promoting lipogenic processes in adipocytes.

Some studies have shown that LS reduces adiposity after dietary intervention, such as LS combined with food restriction [5,32]. Furthermore, several studies administered much lower doses of LS (0.59%) [4,5,33] than that used in the present study (5%). In addition, the initial metabolic condition of the animals in our study may have contributed to a potential lipogenic effect of LS. Thus, our results suggest that LS has few positive effects on the treatment of obesity, hyperglycemia and hyperinsulinemia when these conditions are already present. In addition, our *in vivo *data confirm *in vitro *studies [39] suggesting that, depending on the metabolic condition of the body, leucine exerts a lipogenic effect, possibly by activating mTORC1 in adipocytes [36,37].

Similarly, when insulin-resistant adipocytes were incubated in the presence of leucine and insulin, the recovery of lipogenesis and glucose uptake were observed [39]. From this perspective, these responses support our hypothesis that in the presence of insulin resistance leucine may retrieve the lipogenic effects of insulin, promoting body fat deposition, as proposed by Hinault et al. [39].

Based on our results regarding LS in initially hyperinsulinemic and hyperglycemic animals, no significant effects on serum glucose or insulin levels were observed. These effects agree with data from other studies in which LS did not affect body composition [4,9]. On the other hand, leucine-induced changes in hyperglycemia and hyperinsulinemia have been suggested to depend on changes in adiposity [8].

With regard to hormonal changes, we observed that the type of treatment had different effects on serum TNF-α (ET effect) and adiponectin (LS effect) levels. Whereas LS caused no changes in TNF-α concentration, ET reduced serum TNF-α levels. The concentrations of TNF-α are inversely proportional to the insulin response. Moreover, the lower serum TNF-α levels might be due to reduced adiposity in trained animals [40].

*In vitro *experiments showed that leucine promotes increased synthesis and secretion of adiponectin in 3T3-L1 adipocytes [12,13], and this effect may be mTOR-dependent [41]. Adiponectin is directly associated with improved glucose uptake by increasing insulin sensitivity [42]. However, the increased serum adiponectin level seen in our leucine-supplemented groups did not improve the glucose homeostasis of these animals. Studies suggest that increases in adiponectin concentration may be related to the increase of different isoforms of this adipokine [43], and some of these isoforms are not related to improvement of glucose metabolism or insulin sensitivity [43]. Thus, further studies are required to clarify which isoforms of adiponectin are stimulated by leucine and how this interferes with insulin sensitivity.

Another effect of LS was its ability to reduce the serum concentration of total cholesterol, and this response did not depend on changes in body weight or fat mass. However, leucine was only able to reduce serum cholesterol levels in sedentary animals since the same was not seen in the trained group. This response related to LS agrees with the results of Zhang et al. [8] who observed that the reduction in cholesterol levels was largely independent of leucine-induced changes in adiposity. However, little is known about the molecular mechanisms activated by LS that are able to change cholesterol metabolism.

In conclusion, the present study provides further evidence indicating that the effects of leucine on body composition are highly dependent on the metabolic state of the animals at the beginning of LS. In this respect, several studies have shown no change or a reduction in fat mass when leucine is administered in situations that improve insulin sensitivity, such as food restriction [5] or combined with physical exercise (present study), or simultaneously with a HFD [8,9,44]. On the other hand, a lipogenic effect of leucine treatment is observed when the amino acid is administered to cells previously exposed to situations that induce insulin resistance [39]. Therefore, our findings support a potential lipogenic role of leucine under conditions of preexisting hyperglycemia and hyperinsulinemia as suggested in other studies. The results also showed that ET combined with LS had no additional beneficial effects other than those seen with either treatment alone. Finally, although leucine had no beneficial effects on insulin sensitivity, LS was effective in decreasing the circulating levels of cholesterol and in increasing serum adiponectin concentration.

## List of Abbreviations used

ATGL: adipose triglyceride lipase; CD: control diet; ET: endurance training; ELISA: enzyme-linked immunosorbent assay; EP: epididymal; HDL-c: high-density lipoprotein-cholesterol; HFD: high-fat diet; HSL: hormone sensitive lipase; IL-6: interleukin-6; LS: leucine supplementation; mTOR: mammalian target of rapamycin; REF: reference; RP: retroperitoneal; SC: subcutaneous; TG: triacylglycerol; TNF-α: tumor necrosis factor-alpha.

## Competing interests

The authors declare that they have no competing interests.

## Authors' contributions

For experimental design: FLTL, JDJr, MHFA, MMR, JT; for data collection: FLTL, MHFA, GFRT, MDC, DV, LCP, EMMN; for data analysis: FLTL, JDJr, MHFA, MMR, JT; for drafting the manuscript: FLTL, JDJr, MHFA, MMR, JT. All authors read and approved the final manuscript.
